# Characterization of *Enterobacter cloacae* and *Citrobacter freundii* Species Complex Isolates with Decreased Susceptibility to Cephalosporins from United States Hospitals and Activity of Aztreonam–Avibactam and Comparator Agents (2019–2023)

**DOI:** 10.3390/antibiotics14040382

**Published:** 2025-04-05

**Authors:** Helio S. Sader, Timothy B. Doyle, John H. Kimbrough, Rodrigo E. Mendes, Mariana Castanheira

**Affiliations:** Element Iowa City (JMI Laboratories), 345 Beaver Kreek Centre, Suite A, North Liberty, IA 52317, USA; tim.doyle@element.com (T.B.D.); hank.kimbrough@element.com (J.H.K.); rodrigo.mendes@element.com (R.E.M.); mariana.castanheira@element.com (M.C.)

**Keywords:** aztreonam–avibactam, CRE, metallo-beta-lactamase, NDM, ESBL

## Abstract

Background: *Citrobacter freundii* (CFC) and *Enterobacter cloacae* (ECLC) species complexes represent important causes of hospital-associated infections, frequently are related to outbreaks, and have a great ability to develop antimicrobial resistance. We evaluated a large collection of CFC and ECLC isolates with decreased susceptibility to broad-spectrum cephalosporins (Ceph-DS) from United States (US) hospitals. Methods: A total of 43,325 Enterobacterales (1/patient) were collected in 2019–2023 and susceptibility tested by broth microdilution; among those, 5106 (11.8%) were CFC (*n* = 1374) or ECLC (*n* = 3732). Ceph-DS CFC (*n* = 379) and ECLC isolates (*n* = 1065), defined as isolates with ceftazidime MICs ≥ 16 mg/L and/or cefepime MICs ≥ 2 mg/L, were screened for β-lactamase genes by whole genome sequencing. Results: The most common ESBLs were CTX-M type (*n* = 98; 47.6% of ESBL producers), SHV type (*n* = 94; 45.6%), and OXA type (*n* = 78; 37.9%); ≥2 ESBLs were identified in 65 isolates (31.6%), mainly OXA-1/30 plus a CTX-M. A carbapenemase was identified in 55 of 64 (85.9%) carbapenem-resistant (CB-R) isolates, including KPC type (40 isolates; 62.5% of CB-R) and NDM-1 (16; 23.4% of CB-R). Aztreonam–avibactam was active against 99.6% of Ceph-DS and 100.0% of ESBL producers and CB-R isolates, including NDM producers. Ceftazidime–avibactam and meropenem–vaborbactam were active against 100.0% of ESBL producers (excluding carbapenemase co-producers) and 70.3–71.9% of CB-R isolates. Cefiderocol was active against 82.8% of CB-R isolates but only 46.7% of MBL producers. Conclusions: Aztreonam–avibactam was highly active against cephalosporin-nonsusceptible ECLC and CFC, including MBL producers. The activities of ceftazidime–avibactam, meropenem–vaborbactam, and cefiderocol were compromised against CB-R isolates due to the high frequency of NDM producers.

## 1. Introduction

*Citrobacter freundii* species complex (CFC) and *Enterobacter cloacae* species complex (ECLC) have become relevant among the organisms causing healthcare-associated infections (HAI) due to their ability to develop antimicrobial resistance [1,2]. These organisms are opportunistic pathogens that are responsible for various HAI, including pneumonia, bloodstream infections (BSIs), meningitis, urinary tract infections (UTIs), and skin and skin structure infections (SSSIs). These organisms have also been frequently implicated in hospital outbreaks, especially in neonatal units [3,4,5].

CFC and ECLC belong to the group of Enterobacterales organisms that carry an inducible chromosomal AmpC β-lactamase genes. Treatment with β-lactam agents can cause overexpression of this gene, leading to increasing production of the chromosomal AmpC β-lactamase and resistance to third-generation cephalosporins, piperacillin/tazobactam, and ceftolozane/tazobactam [6,7]. For instance, approximately 20% of the ECLC strains that are initially susceptible in vitro can develop resistance to broad-spectrum β-lactams during antimicrobial treatment [7]. Moreover, the high prevalence of extended-spectrum β-lactamases (ESBL) producers and increasing occurrence of carbapenemase-producing strains among clinical isolates of CFC and ECLC further reduce the availability of antimicrobial agents against invasive and severe infections [8,9,10].

The newer β-lactamase inhibitor combinations (BLICs) ceftazidime–avibactam, meropenem–vaborbactam, and imipenem–relebactam, as well as the siderophore cephalosporin cefiderocol, have demonstrated potent activity and broad coverage against CFC and ECLC from United States (US) hospitals, including isolates resistant to carbapenems. However, resistance to these new agents has emerged in some US regions and new compounds are needed [11]. Aztreonam–avibactam was approved by the European Medicines Agency (EMA) in the European Union in April 2024 (https://www.ema.europa.eu/en/news/new-antibiotic-fight-infections-caused-multidrug-resistant-bacteria; accessed on 1 February 2024) and by the US Food and Drug Administration (FDA) for treatment of complicated intra-abdominal infection on 7 February 2025. Aztreonam–avibactam has demonstrated good activity against multidrug-resistant (MDR) Enterobacterales, including MBL-producing strains [12]. In this investigation, we evaluated the antimicrobial susceptibility and β-lactamase profile of a large collection of CFC and ECLC isolates with decreased susceptibility to broad-spectrum cephalosporins (Ceph-DS) collected from US hospitals.

## 2. Results

**Figure 1:**
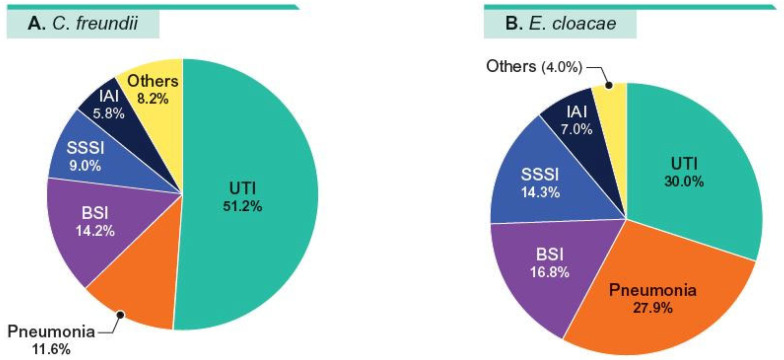
Distribution of organisms by infection type. Abbreviations: UTI—urinary tract infection; BSI—bloodstream infection; SSSI—skin and skin structure infection; IAI—intra-abdominal infection.

CFC and ECLC combined (*n* = 5106) represented 11.8% of Enterobacterales (*n* = 43,235) during the period of the investigation. Among those, 1444 (28.3%) isolates were categorized as Ceph-DS (ceftazidime MIC ≥ 16 mg/L and/or cefepime MIC ≥ 2 mg/L), including 379 CFC (27.6% of total) and 1065 ECLC (28.5% of total; Table 1). The distribution of Ceph-DS isolates by infection type are shown in Figure 1A,B. CFC and ECLC isolates were mainly from patients with UTI (51.2% and 30.0%, respectively), pneumonia (11.6% and 27.9%, respectively), BSI (14.2% and 16.8%, respectively), and SSSI (9.0% and 14.3%).

**Figure 2:**
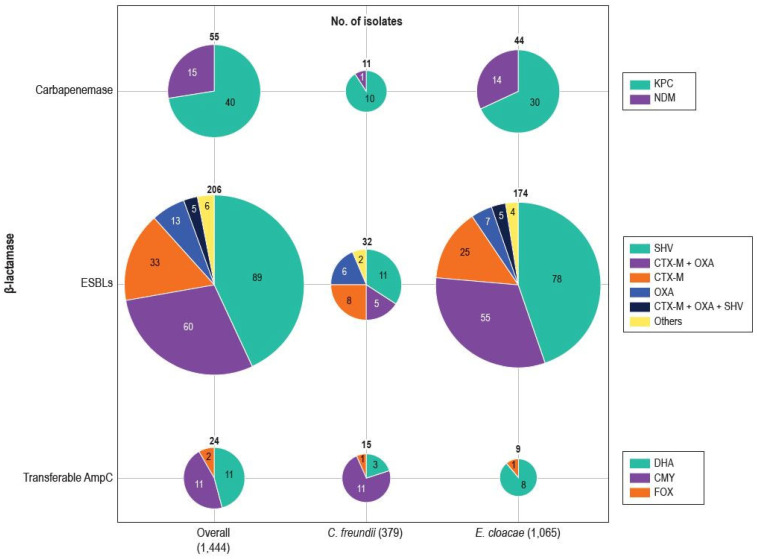
Genes encoding broad-spectrum β-lactamases detected among for 1444 *Enterobacter cloacae* species complex and *Citrobacter freundii* species complex isolates collected in 2019–2023 from United States hospitals and displaying elevated MIC values for ceftazidime (≥16 mg/L) and/or cefepime (≥2 mg/L).

An ESBL gene was detected in 206 isolates (14.3% of Ceph-DS isolates), including 32 (8.4%) CFC and 174 ECLC (16.3%; Figure 2). The most common ESBL encoding genes were *bla*_CTX-M_ (98 isolates; 6.8% of Ceph-DS and 47.6% of ESBL producers) and *bla*_SHV_ (94 isolates; 6.5% of Ceph-DS and 45.6% of ESBL producers). Notably, 33.7% (33/98) of isolates with a *bla*_CTX-M_ also had an ESBL *bla*_OXA_ (Table 1 and Figure 2).

A carbapenemase gene was identified in 55 isolates, which represent 3.8% of Ceph-DS isolates and 85.9% of carbapenem-resistant (CB-R) isolates (Table 1 and Figure 2). The carbapenemase genes identified were *bla*_KPC_ (40 isolates or 62.5% of CB-R isolates) and *bla*_NDM_ (15 isolates or 24.3% of CB-R isolates; Table 1 and Figure 2). Genes encoding transferable AmpC β-lactamases were detected in 24 isolates, with *bla*_CMY_ being the most common among CFC (11 of 15 isolates; 73.3%) and *bla*_DHA_ being the most common among ECLC (8 of 9 isolates; 88.9%; Table 1 and Figure 2). It is noteworthy that transferable AmpC genes were more common among Ceph-DS CFC (4.0%) than Ceph-DS ECLC (0.8%) isolates.

The antimicrobial susceptibility of all CFC and ECLC isolates and respective resistant subsets are displayed in Table 2. Ceftazidime was active against 72.5% of CFC and 70.6% of ECLC (71.1% overall), and cefepime was active against 93.2% of CFC and 89.0% of ECLC (90.1% overall). Meropenem was active against 98.5% of the CFC and ECLC collection and 94.7% of the Ceph-DS isolates overall (Table 2). Aztreonam–avibactam (MIC_50/90_, 0.25/1 mg/L; 99.6% susceptible), ceftazidime–avibactam (MIC_50/90_, 0.5/1 mg/L; 98.2% susceptible), and meropenem–vaborbactam (MIC_50/90_, 0.03/0.06 mg/L; 98.5% susceptible) demonstrated potent activity against Ceph-DS isolates (Table 1).

Aztreonam–avibactam, ceftazidime–avibactam, and meropenem–vaborbactam showed complete activity (100.0% susceptibility) against ESBL producers (excluding carbapenemase co-producers) and transferable AmpC producers. Meropenem was active against 98.8% of ESBL producers (excluding carbapenemase co-producers; MIC_50/90_, 0.03/0.12 mg/L) and 100.0% of transferable AmpC producers (MIC_50/90_, 0.06/0.25 mg/L). In contrast, ceftolozane–tazobactam and piperacillin–tazobactam exhibited limited activity against ESBL producers (excluding carbapenemase co-producers) and transferable AmpC producers (Table 2).

Aztreonam–avibactam (MIC_50/90_, 0.25/1 mg/L) inhibited all carbapenem-resistant (CB-R) isolates at ≤4 mg/L (100.0% susceptible; Table 1). Cefiderocol was the second most active compound against these organisms, with susceptibility rates of 100.0% for CFC and 78.8% for ECLC (82.8% overall). Ceftazidime–avibactam, meropenem–vaborbactam, and imipenem–relebactam were active against 91.7% of CB-R CFC and 65.4% to 67.3% of CB-R ECLC (70.3–71.9% susceptible overall; Table 2).

The highest aztreonam–avibactam MIC value among MBL producers (NDM-1) was only 0.5 mg/L (MIC_50/90_, 0.12/0.5 mg/L; 100.0% susceptible). Cefiderocol (MIC_50/90_, 8/32 mg/L) was active against 46.7% (7/15) of MBL producers, whereas gentamicin and amikacin were active against 86.7% and 73.3% of MBL producers, respectively (Table 2).

Notably, 31.5% of the CFC and ECLC collection exhibited a multidrug-resistant (MDR) (resistance to ≥3 classes) phenotype (Table 3). Moreover, the occurrence of MDR phenotype was >90% among Ceph-DS organisms as well as among organisms producing ESBL, transferable AmpC, or carbapenemases (Table 3). The occurrence of isolates with an extensive drug-resistance (XDR; susceptibility to ≤2 classes) was particularly elevated among ESBL producers (12.6%) and carbapenemase producers (37.8%; Table 3).

There were a total of six (0.1%) aztreonam–avibactam–resistant isolates, which were further identified as *Enterobacter hormaechei* (5 isolates) and *Enterobacter kobei* (1 isolate) by whole genome sequencing (WGS). Each isolate had a unique multi-locus sequence type (MLST). All six isolates were susceptible to meropenem, meropenem–vaborbactam, ceftazidime–avibactam, levofloxacin, gentamicin, and amikacin, and resistant to ceftriaxone, ceftazidime, ceftolozane–tazobactam, and piperacillin–tazobactam. No acquired β-lactamase gene was identified on these isolates, except for a *bla*_OXA-2_ in the *E. kobei*. Also, we did not identify major alterations in the PBP3 gene of these isolates. In contrast, all six isolates showed alterations in OmpC and/or OmpF genes. AmpC gene expression was not evaluated.

## 3. Discussion

The selection of optimal antibiotic therapy for infections caused by CFC, ECLC, and other Enterobacterales species that produce inducible AmpC is complex. AmpC resistance on these organisms can be classified into three categories: (1) inducible chromosomal resistance that emerges during β-lactam treatment, (2) stable derepression due to mutations in ampC regulatory genes, or (3) mediation by transferable (plasmid-mediated) ampC genes [13]. Additionally, resistance to carbapenems can emerge by acquisition of carbapenemase genes or hyperproduction of derepressed AmpC associated with porin alterations that reduce the amount of carbapenem entering the cell [7]. Thus, the use of third-generation cephalosporins or piperacillin–tazobactam are not recommended for isolates that test susceptible to these agents [14]. Moreover, the use of carbapenems has been recommended for the treatment of systemic infections caused by CFC and ECLC isolates that test resistant to third-generation cephalosporin and remain susceptible to carbapenems [14].

In the present investigation, we evaluated the antimicrobial susceptibility and β-lactamase profile of a large collection of CFC and ECLC clinical isolates from US hospitals. Susceptibility rates to third-generation cephalosporins (ceftazidime and ceftriaxone) were around 70% for both organisms, which are comparable to results published by other investigators as well as those previously reported by our group [12,15]. Cefepime remained active against 90.1% of isolates overall, but it is not recommended for the treatment of systemic infections caused by CFC or ECLC isolates resistant to third-generation cephalosporins [14]. Meropenem was active against 98.5% of isolates, indicating that the carbapenems could represent a reliable option for treatment of severe infections caused by these organisms. It is also important to note that the new BLICs, ceftazidime–avibactam and meropenem–vaborbactam, as well as the new siderophore cephalosporin cefiderocol exhibited limited activity against CB-R isolates due to the elevated frequency of NDM producers, whereas aztreonam–avibactam was active against 100.0% of those organisms.

The most common mechanism responsible for decreased susceptibility to broad-spectrum cephalosporins appears to be hyperproduction of chromosomal AmpC, since ESBLs, transferable AmpC, or carbapenemases-encoding genes were not identified in 81.8% (1181/1444) of the Ceph-DS isolates submitted to WGS. The most common acquired β-lactamases causing resistance to third-generation cephalosporins, piperacillin–tazobactam, and ceftolozane–tazobactam were the ESBLs, mainly CTX-M and SHV types. Data on the frequency of occurrence of acquired β-lactamases among CFC, ECLC, or other Enterobacterales species that produce inducible chromosomal AmpC in the US are scarce [12]. There have been sporadic reports of infections and outbreaks caused by ESBL and carbapenemase-producing organisms, mainly from other countries [3,4,5,6,8,16], but investigations on the frequency of ESBLs or acquired carbapenemases are rare. Among ECLC, *E. hormaechei* represent the most common species causing HAI and has been related to various resistance mechanisms [5,16,17,18]. Notably, five of six aztreonam–avibactam-resistant organisms identified in the present study were *E. hormaechei.*

Comparison of the results of this study with those generated in 2017–2019 revealed interesting information. In our previous investigation, we evaluated the β-lactamase profile and antimicrobial susceptibility of 1008 CFC and 2571 ECLC isolates collected from US hospitals in 2017–2019 [12]. Susceptibility rates for ceftriaxone decreased slightly in 2019–2023 (70.2% for CFC and 66.4% for ECLC) compared to 2017–2019 (72.2% for CFC and 68.8% for ECLC). Regarding the β-lactamase profile among Ceph-DS CFC and ECLC, we observed a marked decrease in the frequency of isolates producing ESBLs and carbapenemases in 2019–2023 compared to 2017–2019. The frequency of ESBL producers decreased from 21.1% in 2017–2019 to 14.3% in the present study and the frequency of carbapenemase producers decreased from 6.9% to 3.8%. The frequency of transferable AmpC remained stable, i.e., 1.6% (15/914) in 2017–2019 and 1.7% (24/1444) in 2019–2023 [12]. Importantly, although the overall frequency of carbapenemase producers decreased, the proportion of MBL producers among CB-R CFC and ECLC increased from 6.3% (4/63) to 23.4% (15/64). The rise in MBL producers was due to a significant increase in NDM-1-producing strains and adversely affected the activity of ceftazidime–avibactam, meropenem–vaborbactam, and cefiderocol against CB-R CFC and ECLC.

The fact that we did not evaluate the expression of AmpC genes or porin alterations represents a limitation of the study. However, the evaluation of mutations in genes implicated in AmpC regulation and the porins was beyond the scope of this investigation. The focus of this investigation was to evaluate the β-lactamase profile of contemporary CFC and ECLC and the antimicrobial susceptibility of these organisms to new and recently approved β-lactam agents.

The results of our investigation provide valuable information on the epidemiology of acquired β-lactamases and activities of newer BLICs and cefiderocol against these organisms. The main findings of our investigations were as follows: (1) hyperproduction of chromosomal AmpC remains the main mechanism of resistance to broad-spectrum β-lactams among these organisms; (2) approximately 15% of Ceph-DS organisms produced an ESBL, mainly SHV, CTX-M, and OXA types; (3) the frequency of MBL producers has increased markedly among CB-R organisms in the last few years, with NDM representing almost 30% of the identified carbapenemases; and (4) aztreonam–avibactam was highly active against CFC and ECLC isolates with decreased susceptibility to broad-spectrum cephalosporins and retained complete activity against CB-R isolates, including MBL producers. Notably, the activities of ceftazidime–avibactam, meropenem–vaborbactam, and cefiderocol were compromised against CB-R isolates due to the high frequency of NDM producers.

## 4. Materials and Methods

### 4.1. Organism Collection

A total of 43,325 Enterobacterales isolates were consecutively collected from 80 US medical centers (36 states) in 2019–2023 via a network of medical sites participating in the International Network for Optimal Resistance Monitoring (INFORM) Surveillance Program and sent to Element Iowa City (JMI Laboratories; North Liberty, IA, USA) for susceptibility testing [19]. Each participating center was invited to collect a specific number of consecutive bacterial isolates (any species) each year from patients hospitalized with the following infection types: bloodstream infection, pneumonia, urinary tract infection, skin and skin structure infection, and intra-abdominal infection. The number of isolates varied per infection type. Only isolates determined to be the probable cause of infection by local criteria were included in the investigation.

The magnitude of the INFORM program decreased progressively during the period of the study mainly due to difficulty keeping the participant medical centers enrolled and to enroll new ones. The number of participant centers decreased progressively from 69 in 2019 to 61 in 2023. Consequently, the total number of Enterobacterales collected each year decreased from 9686 in 2019 to 7233 in 2023. Participant medical centers were distributed throughout all US Census Divisions, with the lowest number of medical centers in the East South Central division (varying from 6 centers in 2019 to 3 centers in 2023) and the New England division (5 centers during the entire period of the study) and the highest number of medical centers in the Pacific (7 to 10), Middle Atlantic (8 to 9), and South Atlantic (8 to 9) divisions.

The Enterobacterales collection included 3732 ECLC and 1374 CFC. In this investigation, we evaluated the antimicrobial susceptibility and β-lactamase gene profile of ECLC and CFC isolates with decreased susceptibility to broad-spectrum cephalosporins (Ceph-DS), which were defined as isolates with ceftazidime MICs ≥ 16 mg/L and/or cefepime MICs ≥ 2 mg/L, and included 1065 ECLC and 379 CFC isolates. The CB-R subset includes isolates with MICs ≥ 4 mg/L for imipenem or meropenem [20].

### 4.2. Susceptibility Testing

All isolates were susceptibility tested by the reference broth microdilution method specified by CLSI standards [19] in a central monitoring laboratory (Element Iowa City [JMI Laboratories]; North Liberty, IA, USA). Aztreonam–avibactam was tested with avibactam at a fixed concentration of 4 mg/L. Cefiderocol and imipenem–relebactam were only tested against CRE isolates. Cefiderocol was tested on iron-depleted media [19]. The aztreonam–avibactam breakpoints published by the US FDA (susceptible at ≤4 mg/L and resistant at ≥16 mg/L) were applied (https://www.fda.gov/drugs/development-resources/aztreonam-and-avibactam-injection; accessed on 1 February 2025). CRE was defined as demonstrating imipenem or meropenem MIC values of ≥ 4 mg/L. CLSI and US FDA breakpoints (https://www.fda.gov/drugs/development-resources/antibacterial-susceptibility-test-interpretive-criteria; accessed on 1 February 2025) were applied to the comparator agents where available [20]. Concurrent quality control (QC) testing was performed to ensure proper test conditions and procedures.

MDR phenotype was defined as nonsusceptibility to at least one drug in ≥3 antimicrobial classes and XDR phenotype as susceptibility to ≤2 antimicrobial classes. Ten antimicrobial classes were evaluated and defined as follows: (1) first and second generation cephalosporins and old BLICs (i.e., ampicillin-sulbactam); (2) third to fifth generation cephalosporins and monobactams (aztreonam); (3) carbapenems; (4) BLIC not stable to carbapenemases; (5) BLIC stable to carbapenemases; (6) aminoglycosides; (7) fluoroquinolones; (8) tetracyclines/glycylcycline (tigecycline); (9) folate pathway antagonist (trimethoprim-sulfamethoxazole [TMP-SMX]); and (10) lipopeptide (colistin) [21].

### 4.3. β-Lactamase Screening

All 1065 Ceph-DS ECLC and 379 Ceph-DS CFC isolates were screened for β-lactamase–encoding genes using Next-Generation Sequencing (NGS). Total genomic DNA was extracted using the fully automated Thermo Scientific™ KingFisher™ Flex Magnetic Particle Processor (Cleveland, OH, USA). DNA extracts were quantified using the Qubit™ High Sensitivity DS-DNA assay (Invitrogen, ThermoFisher Inc., Waltham, MA, USA) and normalized to 0.2 ng/µL. A total of 1 ng high-quality genomic DNA was used as input material for library construction using the Nextera XT™ DNA library preparation kit (Illumina, San Diego, CA, USA). Libraries were normalized using the bead-based normalization procedure (Illumina) and sequenced on MiSeq. The generated FASTQ files were assembled using SPAdes Assembler Version 3.15.3 and subjected to proprietary software (Element Iowa City [JMI Laboratories]) for screening of β-lactamase genes [22]. An in-house proprietary bioinformatic pipeline and an Element Iowa City-curated resistance gene database (Version 3; uses Python v2.7.9, SPAdes v3.15.3, and BBMap v36.x) based on the NCBI Bacterial Antimicrobial Resistance Reference Gene Database (https://www.ncbi.nlm.nih.gov/bioproject/PRJNA313047; accessed on 1 February 2024) was used for the in silico analysis [23].

## Figures and Tables

**Table 1:** Carbapenemases, extended-spectrum β-lactamases (ESBLs), and transferable AmpC enzymes, identified among *C. freundii* species complex and *E. cloacae* species complex isolates with decreased susceptibility to broad-spectrum cephalosporins.

Beta-Lactamase/Organism	No. of Isolates
**Carbapenemases**	**55**
*C. freundii*	11
KPC-18	1
KPC-2	7
KPC-3	2
NDM-1	1
*E. cloacae*	44
KPC-2	14
KPC-3	12
KPC-4	1
KPC-6	2
KPC-10, KPC-6	1
NDM-1	14
**ESBL**	**206**
*C. freundii*	32
CTX-M-1	1
CTX-M-3	2
CTX-M-3, SHV-12	1
CTX-M-13	1
CTX-M-15	4
CTX-M-15, OXA-1/30	5
OXA-1/30	5
OXA-4, GES-7	1
OXA-45	1
SHV-5	1
SHV-7	3
SHV-12	7
*E. cloacae*	174
CTX-M-3	7
CTX-M-9	3
CTX-M-14	1
CTX-M-15	13
CTX-M-15, OXA-1/30	55
CTX-M-15, OXA-1/30, SHV-12	5
CTX-M-15, SHV-12	1
CTX-M-88	1
OXA-1/30	6
OXA-1/30, SHV-12	1
OXA-1/30, SHV-7	1
OXA-17	1
SHV-5	1
SHV-7	8
SHV-7-like	1
SHV-12	65
SHV-30	3
TEM-19	1
**Transferable AmpC**	**24**
*C. freundii*	15
CMY-159	2
CMY-179	3
CMY-180	4
CMY-181	2
DHA-1	3
FOX-5-like	1
*E. cloacae*	9
DHA-1	8
FOX-5	1

**Table 2:** Antimicrobial susceptibility of *Enterobacter cloacae* species complex and *Citrobacter freundii* species complex from United States hospitals (2019–2023).

Antimicrobial	*C. freundii* (No.)		*E. cloacae* (No.)		All (No.)	
	MIC_50/90_	%S ^a^	MIC_50/90_	%S ^a^	MIC_50/90_	%S ^a^
All organisms	(*n* = 1374)		(*n* = 3732)		(*n* = 5106)	
Aztreonam–avibactam ^a^	0.06/0.25	100.0	0.06/0.5	99.8	0.06/0.5	99.9
Ceftazidime–avibactam	0.12/0.5	99.9	0.25/0.5	99.4	0.25/0.5	99.5
Meropenem–vaborbactam	0.03/0.03	99.9	0.03/0.03	99.5	0.03/0.03	99.6
Ceftolozane–tazobactam	0.25/16	77.1	0.25/16	77.6	0.25/16	77.5
Piperacillin–tazobactam	4/128	72.1	2/128	71.9	2/128	72.0
Aztreonam	0.25/>16	72.4	0.12/>16	71.4	0.12/>16	71.7
Ceftriaxone	0.25/>8	70.2	0.25/>8	66.4	0.25/>8	67.4
Ceftazidime	0.5/>32	72.5	0.5/>32	70.6	0.5/>32	71.1
Cefepime	0.06/2	93.2	0.06/4	89.0	0.06/2	90.1
Meropenem	0.03/0.06	98.9	0.03/0.12	98.3	0.03/0.06	98.5
Ertapenem ^b^	0.015/0.25	97.3	0.06/1	86.8	0.06/1	89.6
Levofloxacin	0.06/1	87.8	0.03/0.5	93.0	0.06/0.5	91.6
Gentamicin	0.5/1	93.7	0.25/0.5	96.1	0.25/0.5	95.5
Amikacin	2/2	97.7	1/2	98.8	1/2	98.5
Tobramycin	0.5/2	92.4	0.5/0.5	95.0	0.5/1	94.3
Colistin	0.25/0.5	99.7 ^c^	0.25/>8	79.6 ^c^	0.25/>8	85.1 ^c^
Ceph-DS organisms ^d^	(*n* = 379) ^d^		(*n* = 1065) ^d^		(*n* = 1444) ^d^	
Aztreonam–avibactam ^a^	0.25/0.5	100.0	0.5/1	99.4	0.25/1	99.6
Ceftazidime–avibactam	0.5/1	99.5	0.5/1	97.7	0.5/1	98.2
Meropenem–vaborbactam	0.03/0.03	99.7	0.03/0.06	98.2	0.03/0.06	98.5
Ceftolozane–tazobactam	16/>16	17.4	8/>16	21.9	8/>16	20.7
Piperacillin–tazobactam	128/>128	9.2	64/>128	9.1	128/>128	9.2
Aztreonam	>16/>16	2.9	>16/>16	2.5	>16/>16	2.6
Ceftriaxone	>8/>8	0.3	>8/>8	0.4	>8/>8	0.3
Ceftazidime	>32/>32	1.8	>32/>32	1.1	>32/>32	1.3
Cefepime	1/8	75.2	2/32	61.3	2/32	65.0 ^g^
Meropenem	0.06/0.12	96.3	0.06/0.25	94.2	0.06/0.25	94.7
Ertapenem ^b^	0.25/0.5	91.0	0.5/>2	60.5	0.5/2	68.2
Levofloxacin	0.12/2	76.3	0.06/2	82.7	0.06/2	81.0
Gentamicin	0.5/8	88.9	0.25/4	89.5	0.25/4	89.3
Amikacin	2/4	93.9	1/2	96.8	1/2	96.1
Tobramycin	0.5/8	85.9	0.5/8	85.8	0.5/8	85.9
Colistin	0.25/0.5	98.9 ^c^	0.25/>8	83.5 ^c^	0.25/>8	87.6 ^c^
ESBL producers (excluding CBase) ^e^	(*n* = 27)		(*n* = 142)		(*n* = 169)	
Aztreonam–avibactam ^a^	0.12/1	100.0	0.12/0.5	100.0	0.12/0.5	100.0
Ceftazidime–avibactam	0.5/1	100.0	0.25/1	100.0	0.5/1	100.0
Meropenem–vaborbactam	0.03/0.03	100.0	0.03/0.06	100.0	0.03/0.06	100.0
Ceftolozane–tazobactam	1/>16	63.0	1/>16	66.9	1/>16	66.3
Piperacillin–tazobactam	8/>128	55.6	16/>128	46.8	16/>128	48.2
Aztreonam	>16/>16	7.4	>16/>16	3.5	>16/>16	4.1
Ceftriaxone	>8/>8	0.0	>8/>8	1.4	>8/>8	1.2
Ceftazidime	>32/>32	11.1	>32/>32	7.0	>32/>32	7.7
Cefepime	16/>32	29.6	16/>32	23.9	16/>32	24.9
Meropenem	0.03/0.12	100.0	0.03/0.12	98.6	0.03/0.12	98.8
Ertapenem ^b^	0.03/0.25	100.0	0.5/1	81.1	0.12/1	85.5
Levofloxacin	2/16	29.6	1/16	48.6	1/16	45.6
Gentamicin	16/>16	48.1	16/>16	43.0	16/>16	43.8
Amikacin	2/16	74.1	1/8	87.3	2/8	85.2
Tobramycin	8/>16	33.3	8/>16	29.6	8/>16	30.2
Colistin	0.25/0.5	100.0 ^c^	0.25/>8	87.3 ^c^	0.25/8	89.3 ^c^
Transferable AmpC producers	(*n* = 15)		(*n* = 9)		(*n* = 24)	
Aztreonam–avibactam ^a^	0.12/0.5	100.0	0.25/-	100.0	0.25/1	100.0
Ceftazidime–avibactam	0.25/4	100.0	0.5/-	100.0	0.5/4	100.0
Meropenem–vaborbactam	≤0.015/0.25	100.0	0.03/-	100.0	0.03/0.12	100.0
Ceftolozane–tazobactam	16/>16	40.0	0.5/-	55.6	8/>16	45.8
Piperacillin–tazobactam	32/>128	26.7	16/-	44.4	32/>128	33.3
Aztreonam	>16/>16	13.3	16/-	44.4	>16/>16	25.0
Ceftriaxone	>8/>8	0.0	8/-	0.0	>8/>8	0.0
Ceftazidime	>32/>32	6.7	>32/-	11.1	>32/>32	8.3
Cefepime	1/32	66.7	0.12/-	66.7	1/32	66.7
Meropenem	0.03/0.12	100.0	0.12/-	100.0	0.06/0.25	100.0
Ertapenem ^b^	0.25/-	80.0	0.5/-	60.0	0.5/1	70.0
Levofloxacin	0.5/32	53.3	1/-	44.4	0.5/8	50.0
Gentamicin	1/>16	73.3	0.5/-	77.8	0.5/>16	75.0
Amikacin	2/16	86.7	2/-	77.8	2/16	83.3
Tobramycin	1/16	60.0	0.5/-	66.7	1/16	62.5
Colistin	0.25/0.25	100.0 ^c^	0.25/-	66.7	0.25/>8	87.5
Carbapenem-resistant ^f^	(*n* = 12)		(*n* = 52)		(*n* = 64)	
Aztreonam–avibactam ^a^	0.12/0.5	100.0	0.25/1	100.0	0.25/1	100.0
Ceftazidime–avibactam	1/4	91.7	2/>32	67.3	1/>32	71.9
Meropenem–vaborbactam	0.03/1	91.7	0.06/32	65.4	0.06/32	70.3
Imipenem–relebactam ^g^	0.12/0.5	91.7	0.25/>8	67.3	0.25/>8	71.9
Cefiderocol ^f^	0.12/1	100.0	2/16	78.8	1/16	82.8
Levofloxacin	8/32	8.3	1/32	40.4	2/32	34.4
Gentamicin	8/>16	25.0	0.5/16	73.1	0.5/16	64.1
Amikacin	8/16	41.7	2/8	82.7	2/16	75.0
Colistin	0.25/0.25	100.0 ^c^	0.25/>8	86.5 ^c^	0.25/>8	89.1 ^c^
MBL producers	(*n* = 1)		(*n* = 14)		(*n* = 15)	
Aztreonam–avibactam ^a^		100.0	0.12/0.25	100.0	0.12/0.5	100.0
Ceftazidime–avibactam		0.0	>32/>32	0.0	>32/>32	0.0
Meropenem–vaborbactam		0.0	16/>32	0.0	32/>32	0.0
Imipenem–relebactam ^g^		0.0	8/>8	0.0	8/>8	0.0
Aztreonam		100.0	>16/>16	7.1	>16/>16	13.3
Cefiderocol ^g^		100.0	8/32	42.9	8/32	46.7
Levofloxacin		0.0	1/16	35.7	2/32	33.3
Gentamicin		0.0	0.5/2	92.9	0.5/4	86.7
Amikacin		100.0	4/16	71.4	4/16	73.3
Colistin		100.0 ^c^	0.25/0.25	92.9 ^c^	0.25/0.25	93.3 ^c^
Ceph-DS isolates with no ESBL, no transferable AmpC, and no carbapenemase	(*n* = 324)		(*n* = 857)		(*n* = 1181)	
Aztreonam–avibactam ^a^	0.25/0.5	100.0	0.5/1	99.3	0.5/1	99.5
Ceftazidime–avibactam	0.5/1	100.0	0.5/1	99.3	0.5/1	99.5
Meropenem–vaborbactam	0.03/0.03	100.0	0.03/0.06	100.0	0.03/0.03	100.0
Ceftolozane–tazobactam	16/>16	14.5	8/>16	15.6	8/>16	15.3
Piperacillin–tazobactam	128/>128	5.6	64/>128	3.3	128/>128	3.9
Aztreonam	>16/>16	1.9	>16/>16	2.1	>16/>16	2.0
Ceftriaxone	>8/>8	0.3	>8/>8	0.2	>8/>8	0.3
Ceftazidime	>32/>32	1.2	>32/>32	0.2	>32/>32	0.5
Cefepime	1/4	82.7	2/4	71.1	1/4	74.3
Meropenem	0.06/0.06	99.7	0.06/0.25	98.5	0.06/0.12	98.8
Ertapenem ^b^	0.25/0.5	93.7	0.5/2	61.4	0.5/2	69.6
Levofloxacin	0.12/1	84.3	0.06/0.5	91.9	0.06/1	89.8
Gentamicin	0.5/1	96.0	0.25/0.5	98.7	0.25/0.5	98.0
Amikacin	2/2	98.5	1/2	99.5	1/2	99.2
Tobramycin	0.5/1	94.4	0.5/0.5	98.6	0.5/1	97.5
Colistin	0.25/0.5	98.8 ^c^	0.25/>8	83.0 ^c^	0.25/>8	87.4 ^c^

^a^ % susceptible per CLSI criteria (2025), except for aztreonam–avibactam where US FDA breakpoints were applied. ^b^ Ertapenem alone was tested against isolates from a subset of medical centers, including 551 CFC (167 Ceph-DS) and 1543 ECLC (496 Ceph-DS). ^c^ % of isolates with colistin MIC ≤ 2 mg/L, i.e., intermediate by CLSI (2025) and susceptible per EUCAST (2025). ^d^ Isolates resistant to ceftazidime (MIC ≥ 16 mg/L) and/or nonsusceptible to cefepime (MIC > 2 mg/L). ^e^ Include only ESBL-producing isolates with ceftazidime MIC ≥ 16 mg/L and/or cefepime MIC ≥ 2 mg/L. ^f^ Include isolates with MIC ≥ 4 mg/L for imipenem or meropenem. ^g^ Imipenem–relebactam and cefiderocol were tested against carbapenem-resistant isolates only.

**Table 3:** Occurrence of multidrug-resistance (MDR) and extensive drug-resistance (XDR) phenotypes among the organism subsets.

Organism Subset (No.)	MDR Rate	XDR Rate
All CFC and ECLC (5106)	31.5%	0.5%
Ceph-DS isolates (1444)	96.8%	2.6%
ESBL producers (206)	97.1%	12.6%
Transferable AmpC producers (24)	91.7%	0.0%
Carbapenemase producers (74)	100.0%	37.8%

Abbreviations: CFC—*Citrobacter freundii* species complex; ECLC—*Enterobacter cloacae* species complex; Ceph-DS—decreased susceptibility to broad-spectrum cephalosporins.

## Data Availability

Data is not available due to privacy. The bacterial isolates are still involved in ongoing investigations.
